# Supplementary material for: Local network connectivity optimization: an evaluation of heuristics applied to complex spatial networks, a transportation case study, and a spatial social network
Source: PeerJ Comput Sci. 2021 Jun 18;7:e605. doi: 10.7717/peerj-cs.605 (PMC8237331; doi:10.7717/peerj-cs.605)
Supplement: Supplemental Information 1 [file peerj-cs-07-605-s001.pdf]

Supporting Information

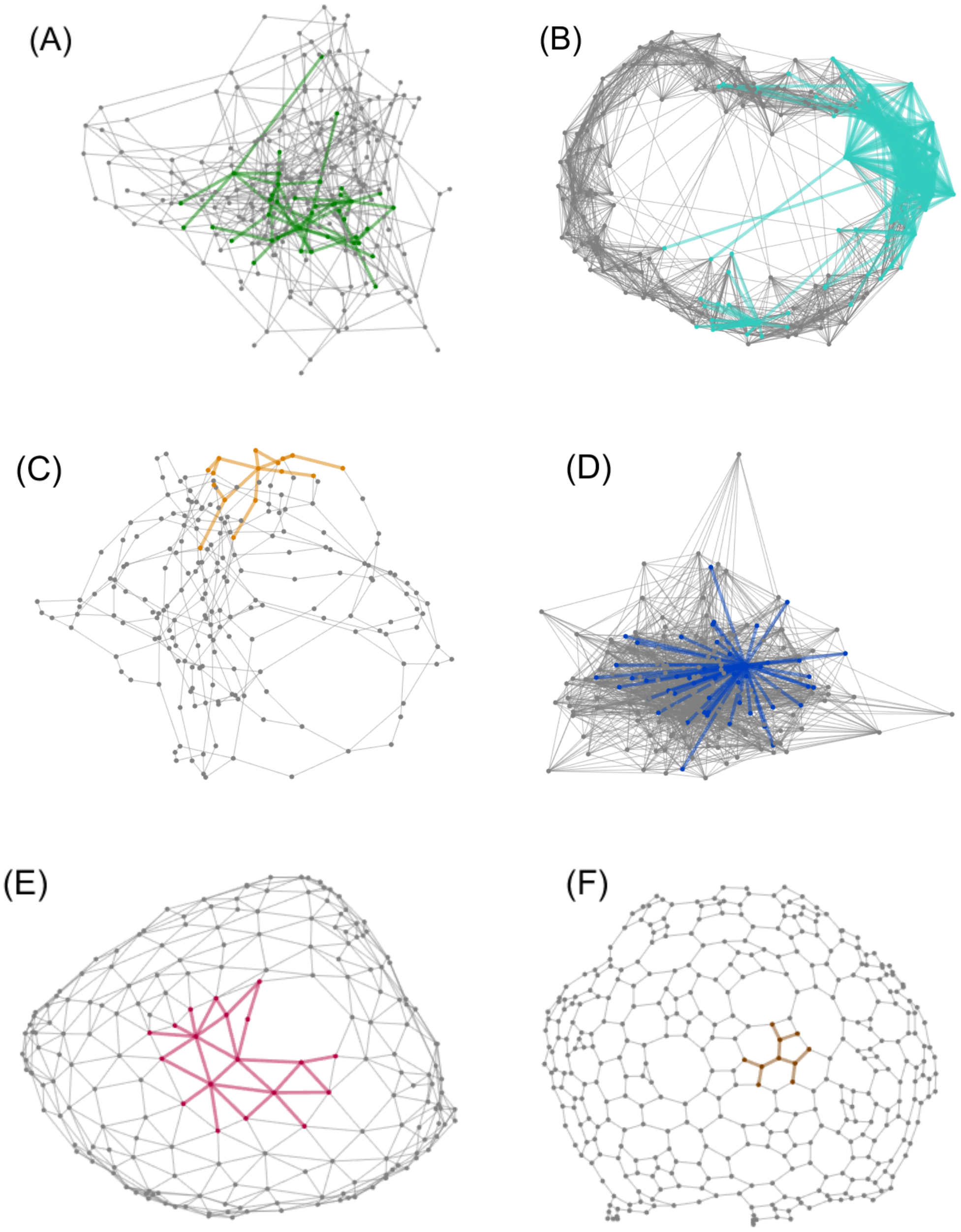

Figure S.1: Examples of the random graphs ($N = 250$) used for the analysis: (A) Erdös-Rényi network, (B) Watts-Strogatz network, (C) Barabási and Albert network, and (D) Klemm and Eguílez network, (E) Delaunay network, and (F) Voronoi network. The colored subgraph represents the close nodes to a random focal node with high connectivity and the edge weights (distances) are not to scale for a clearer visualization.

Local search selection criteria		
Nodal characteristics	distance from focal node $d(i, F)$	degree centrality $C_i^D = \sum_j A(i,j)$
	closeness centrality $C_i^C = 1/\sum_j d(i,j)$	betweenness centrality $C_i^B = \sum_{j \neq i \neq k} \frac{\sigma_{jk}(i)}{\sigma_{jk}}$
	eigenvector centrality $C_i^E = \frac{1}{\lambda} \sum_j A(i,j) x_j$	pagerank centrality $C_i^P = \alpha_P \sum_j A(i,j) \frac{x_j}{\sum_i A(i,j)} + \frac{1-\alpha_P}{N}$
	weighted clustering coefficient $c_i^w = \frac{1}{(C_i^D - 1)\sum_j a_{ij} w_{ij}} \sum_{j,h} \frac{(w_{ij} + w_{ih})}{2} a_{ij} a_{ih} a_{jh}$	
Clusters	hierarchical clusters	
	network-constrained clusters	
	network modularity	

Table S.1: Node characteristics used for the neighborhood search and their formulations.

Measures of network complexity. To evaluate the complexity of the random Voronoi networks and random Delaunay networks we use the following measures that capture the random, scale-free, and small-world features of complex networks: average degree, average path length, weighted clustering coefficient, proximity ratio, global efficiency, and power law (see Table S.2 for the mathematical formulations of these characteristics). General measures of connectivity and network topology (for connected graphs) include the mean degree and the average path length, which refers to the average number of edges within the shortest path for all pairs of nodes in a network [1]. The weighted clustering coefficient of a node is the ratio of the node degree and the total number of possible edges for a node in the network [2]. The global version of this measure is the average of the node weighted clustering coefficients and provides an estimate of small-world-ness [3]. The proximity ratio is the ratio of the following ratios: (i) the average weighted clustering coefficient and the average path length and (ii) the average weighted clustering coefficient for a completely random network of the same size and the average path length for that random network of the same size. This provides a measure of the small-world-ness of networks, with $S = 1$ for random networks and $S \gg 1$ for small-world networks [4]. Another measure of small-world-ness is average efficiency which is the average of the inverses of the network's shortest paths and captures the network's ability to exchange information between nodes [5]. If the degree distribution of the nodes follows a power law, then the network is said to be scale-free, i.e. random with some highly connected nodes [6].

Measures of network complexity	
mean degree $\overline{C_D} = \frac{\sum_i C_i^D}{N}$	average path length $L = \frac{1}{N(N-1)} \sum_i \sum_{j>i} d(i,j)$
weighted clustering coefficient $\overline{C} = \frac{1}{N} \sum_i c_i^w$	proximity ratio $S = \frac{\overline{C}/L}{\overline{C_r}/L_r}$
power law $P(n) \approx n^{-\gamma}$	global efficiency $E_G = \frac{E}{E_r}, E = \frac{1}{N(N-1)} \sum_i \sum_{j>i} \frac{1}{d(i,j)}$

Table S.2: The measures used to evaluate network complexity.

	Delaunay			Voronoi		
N	500	1000	2000	500	1000	2000
$\overline{C_D}$	4.708	5.392	5.208	2.840	2.900	2.938
L	9	12	17	21	29	40
$\overline{C}$	0.003	0.003	0.003	0.006	0.003	0.003
S	1.005	0.991	0.980	0.845	0.091	0.089
γ	1.667	1.800	3.000	837	1784	1907
E_G	0.0724	0.0523	0.0385	0.0328	0.0238	0.0177

Table S.3: The average characteristics of 1000 random Delaunay and Voronoi networks.

	Suburban school network						Rural school network			
	Elementary			Middle			Elementary			
	SE(1)	SE(2)	SE(3)	SM(1)	SM(2)	SM(3)	RE(1)	RE(2)	RE(3)	RE(4)
N	3952	4222	5149	7750	4895	8059	1727	2160	1539	1996
N_C	1772	1807	1077	4614	1198	2210	416	330	363	735
N_D	2180	2415	4072	3136	3697	5849	1311	1830	1176	1261
L_F	50	96	194	110	43	103	51	95	50	52
$\overline{C_D}$	2	2	2	2	2	2	2	2	2	2
L	80	133	183	135	72	123	77	88	71	82
γ	1179	1300	1604	2353	1432	2427	761	967	679	891
E_G	0.0060	0.0060	0.0045	0.0052	0.0074	0.0056	0.0078	0.0080	0.0104	0.0092

Table S.4: The network characteristics of the street networks around the schools used for the study. The average weighted clustering coefficient $\overline{C}$ was not calculated since triplets were uncommon in these networks and neither was the proximity ratio S since it depends on $\overline{C}$.

Parameter		Description	Values
	N	Number of nodes	500, 1000, 2000
Erdös-Rényi graphs	p	connection probability	0.01
Watts-Strogatz graphs	p_W	rewiring probability	0.01
	k_L	initial node degree	10
Barabási and Albert graphs	m_0	connected network size	10
	m	degree of new nodes	$m0$
Klemm and Eguílez graphs	m_0	connected network size	10
	p_S	node selection probability	0.1
	p	connection probability	0.01
Delaunay random graphs	p_R	edge removal probability	0.1
Voronoi random graphs	p_R	edge removal probability	0.1

Table S.5: The random network parameters and values used for the analysis. Following [6], the degree of new nodes for the Barabási and Albert graphs were equal to the initial network size ($m = m_0$).

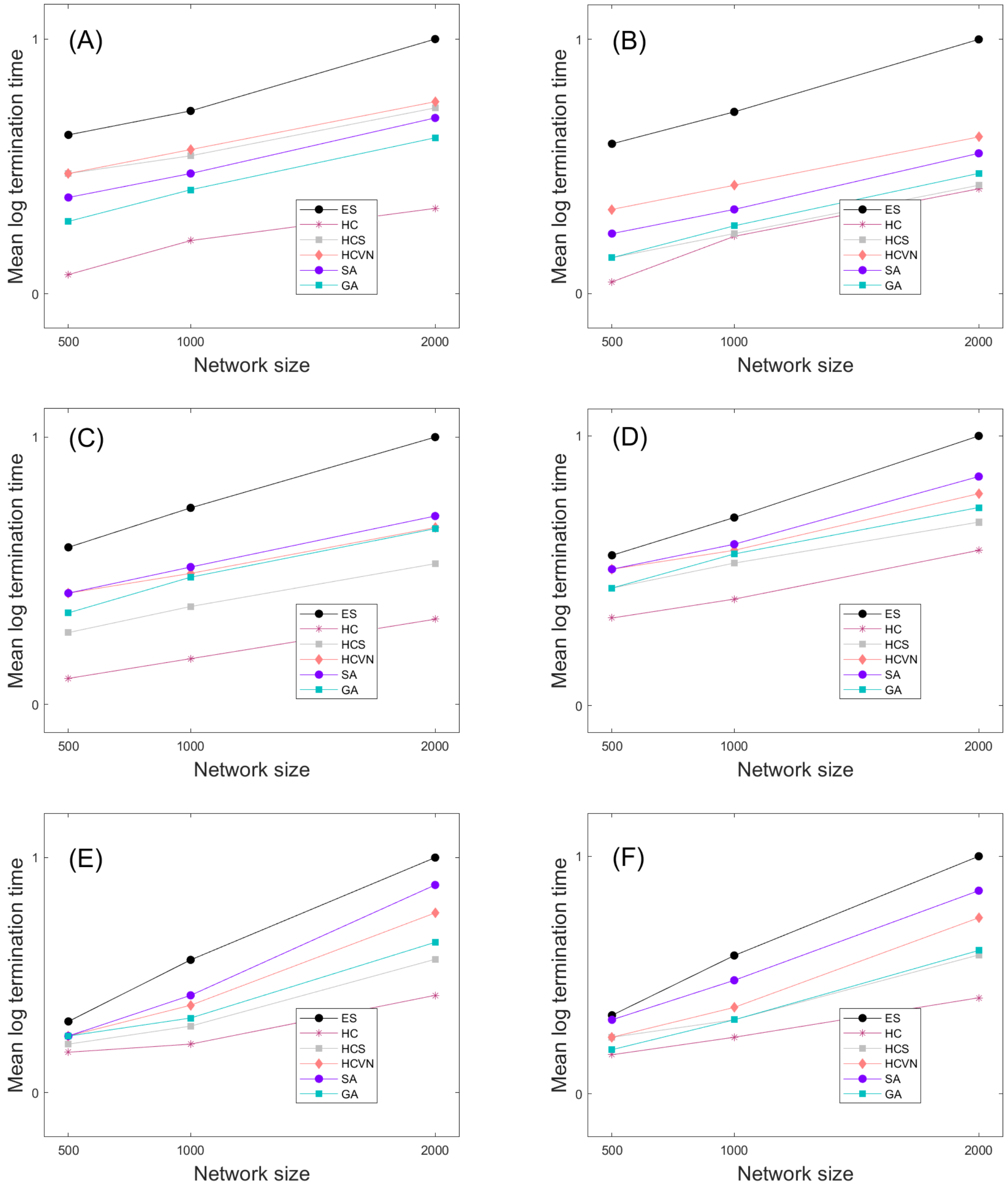

Figure S.2: The termination times for the heuristics applied to the random networks: (a) Erdös-Rényi networks, (b) Watts-Strogatz networks, (c) Barabási and Albert networks, and (d) Klemm and Eguílez networks, (e) Delaunay networks, and (f) Voronoi networks. These are average times for 1000 random restarts for optimization applied to 1000 random network of each type and size. The times are scaled by the exhaustive search time and log transformed for easier interpretation.

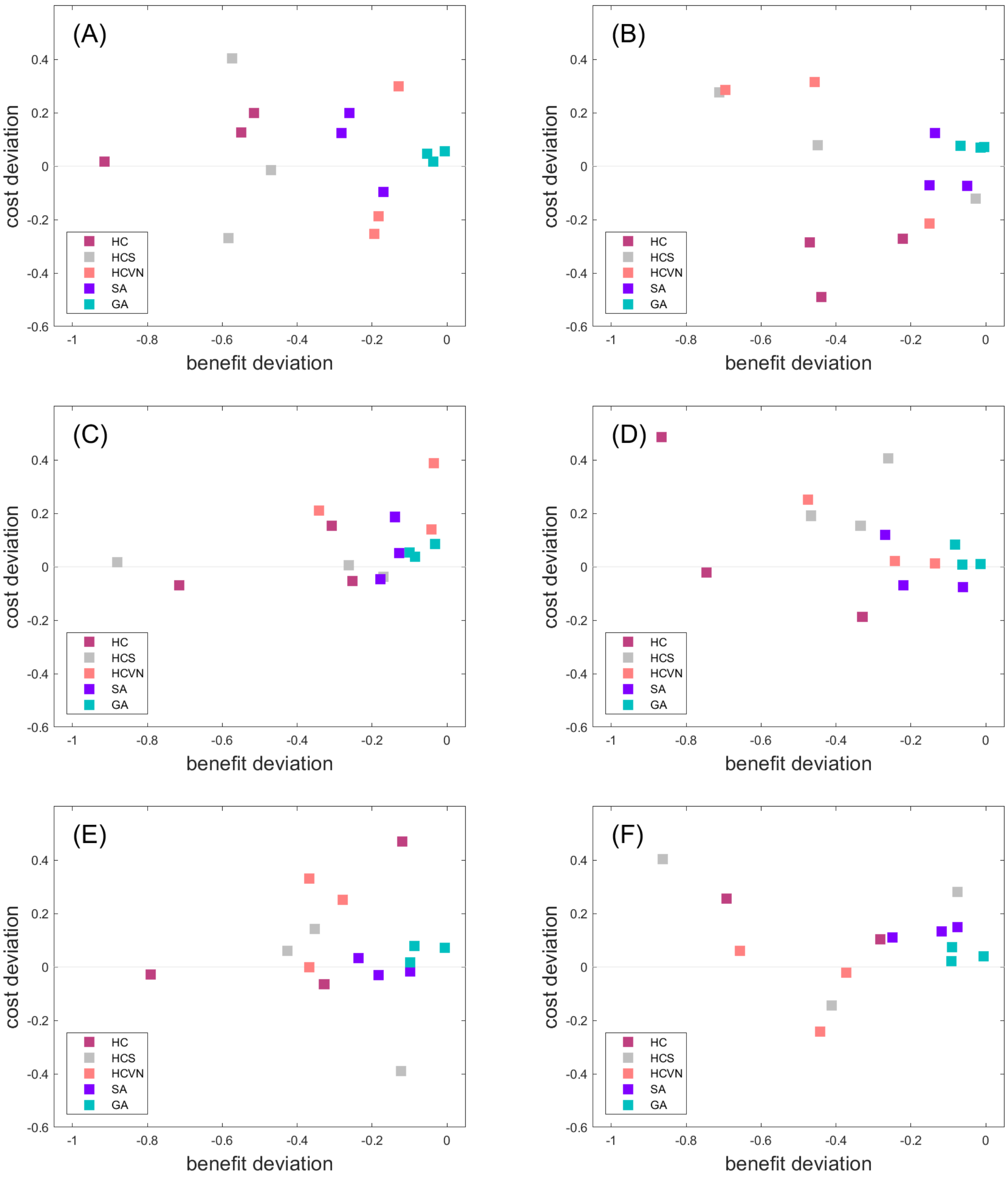

Figure S.3: The cost and benefit deviations for the heuristics applied to the random networks: (a) Erdös-Rényi networks, (b) Watts-Strogatz networks, (c) Barabási and Albert networks, and (d) Klemm and Eguílez networks, (e) Delaunay networks, and (f) Voronoi networks. Each point represents the results for a given method and network size ($N = 500, 1000, 2000$). The costs and benefits were scaled by the results from the exhaustive search, where a longer connection length is a positive cost deviation and a shorter connection is a negative cost deviation. These are average times for 1000 random restarts for optimization applied to 1000 random network of each type and size.

Algorithm 1
Exhaustive search pseudocode

```
for i in N_D do                                        ▷ Select a distant node.
    for j in N_C do                                    ▷ Select each close node.
        if d(i,j) + d(j,S) < D then                    ▷ If node will be within the distance.
            C(i,j) = d(i,j)                            ▷ Calculate the cost of the connection, i.e. the length.
            for k in N_D do                            ▷ Select each distant node.
                if d(k,i) + d(i,j) + d(j,S) < D then   ▷ Calculate the distance to the focal node.
                    k ∈ N'_C                           ▷ If node is within the distance assign it to the new close set.
                end if
            end for
            B(i,j) = |N'_C|                            ▷ Calculate the number of new close nodes.
        end if
    end for
end for
```

Algorithm 2
Hill climbing

```
randomly select $i$ from $N_D$
randomly select $j$ from $N_C$
$C(i,j) = d(i,j)$
for k in $N_D$ do
    if $d(k,i) + d(i,j) + d(j,F) < D$ then
        $k \in N'_C$
    end if
end for
$B(i,j) = |N'_C|$
$O^0 = \alpha C(i,j) + \beta B(i,j)$
$t = 1$
while $O^t \neq O^{t-1}$ do
    $N_i^D$ = neighbors of $i$ in $N_D$
    $N_j^C$ = neighbors of $j$ in $N_C$
    for m in $N_i^D$ do
        for n in $N_j^C$ do
            $C(m,n) = d(m,n)$
            for k in $N_D$ do
                if $d(k,m) + d(m,n) + d(n,F) < D$ then
                    $k \in N'_C$
                end if
            end for
            $B(m,n) = |N'_C|$
        end for
    end for
    $O^t = \max_{(m,n)}(\alpha C(m,n) + \beta B(m,n))$
    $t = t + 1$
end while
```

Algorithm 3

Stochastic hill climbing

```
randomly select i from N_D
randomly select j from N_C
C(i,j) = d(i,j)
for k in N_D do
    if d(k,i) + d(i,j) + d(j,F) < D then
        k ∈ N'_C
    end if
end for
B(i,j) = |N'_C|
O^0 = αC(i,j) + βB(i,j)
t = 1
while O^t ≠ O^{t-1} do
    N_i^D = neighbors of i in N_D
    N_j^C = neighbors of j in N_C
    for m in N_i^D do
        for n in N_j^C do
            C(m,n) = d(m,n)
            for k in N_D do
                if d(k,m) + d(m,n) + d(n,F) < D then
                    k ∈ N'_C
                end if
            end for
            B(m,n) = |N'_C|
        end for
    end for
    O^t = αC(i,j) + βB(i,j) with probability(i,j) = (αC(i,j) + βB(i,j)) / Σ_(m,n)(αC(m,n) + βB(m,n))
    t = t + 1
end while
```

Algorithm 4
Hill climbing with a variable neighborhood

set n_{max}
randomly select i from N_D
randomly select j from N_C
$C(i,j) = d(i,j)$
for k **in** N_D **do**
    if $d(k,i) + d(i,j) + d(j,F) < D$ **then**
        $k \in N'_C$
    end if
end for
$B(i,j) = |N'_C|$
$O^0 = \alpha C(i,j) + \beta B(i,j)$
$\eta = 1$
$t = 1$
while $\eta < \eta_{max}$ **do**
    N_i^D = neighbors of i in N_D
    N_j^C = neighbors of j in N_C
    for m **in** N_i^D **do**
        for n **in** N_j^C **do**
            $C(m,n) = d(m,n)$
            for k **in** N_D **do**
                if $d(k,m) + d(m,n) + d(n,F) < D$ **then**
                    $k \in N'_C$
                end if
            end for
            $B(m,n) = |N'_C|$
        end for
    end for
    $O^t = \max_{(m,n)}(\alpha C(m,n) + \beta B(m,n))$
    if $O^t > O^{t-1}$ **then**
        $\eta = 1$
    else
        $\eta = \eta + 1$
    end if
    $t = t + 1$
end while

Algorithm 5
Simulated annealing

```
randomly select i from N_D
randomly select j from N_C
C(i,j) = d(i,j)
for k in N_D do
    if d(k,i) + d(i,j) + d(j,F) < D then
        k ∈ N'_C
    end if
end for
B(i,j) = |N'_C|
O^0 = αC(i,j) + βB(i,j)
t = 1
while O^t ≠ O^{t-1} do
    N_i^D = neighbors of i in N_D
    N_j^C = neighbors of j in N_C
    for m in N_i^D do
        for n in N_j^C do
            C(m,n) = d(m,n)
            for k in N_D do
                if d(k,m) + d(m,n) + d(n,F) < D then
                    k ∈ N'_C
                end if
            end for
            B(m,n) = |N'_C|
            O(m,n) = αC(m,n) + βB(m,n)
        end for
    end for
    if max_{(m,n)} O(m,n) > O^{t-1} then
        O^t = O(m,n)
    else
        O^t = O(m,n) with probability(m,n) = exp(-(O^{t-1} - O(m,n))/t)
    end if
    t = t + 1
end while
```

Algorithm 6
Genetic algorithm

```
μ = mutation rate
s = selection coefficient
P = randomly selected population
Chromosome(i) = (1...1)
Chromosome(j) = (1...1)
for (i, j) in P do
    C(i, j) = d(i, j)
    for k in N_D do
        if d(k, i) + d(i, j) + d(j, F) < D then
            k ∈ N'_C
        end if
    end for
    B(i, j) = |N'_C|
    O(i, j)^0 = αC(i, j) + βB(i, j)
end for
t = 1
while max_(i,j) O(i, j)^t ≠ max_(i,j) O(i, j)^(t−1) do
    f(i, j) = O(i, j) / Σ_(i,j) O(i, j)
    populate P with (i, j) with probability(i, j) = (s ∗ f(i, j) + (1 − s)) / Σ_(m,n)(s ∗ f(m, n) + (1 − s))
    cycle crossover
    for k in P do
        for ℓ in chromosome(k) do
            if random number ≤ μ then
                gene(k, ℓ) = gene(k, ℓ) + 1
            end if
        end for
    end for
    for (i, j) in P do
        randomly select m from N_i^D with probability gene(i, m)/Σ_k gene(i, k)
        randomly select n from N_j^C with probability gene(j, n)/Σ_k gene(j, k)
        C(m, n) = d(m, n)
        for k in N_D do
            if d(k, m) + d(m, n) + d(n, F) < D then
                k ∈ N'_C
            end if
        end for
        B(m, n) = |N'_C|
        O(m, n)^t = αC(m, n) + βB(m, n)
    end for
    t = t + 1
end while
```

References

[1] Albert, R. & Barabási, A. Statistical mechanics of complex networks. *Reviews of Modern Physics* **74**, 47–97 (2002).

[2] Luce, R. & Perry, A. A method of matrix analysis of group structure. *Psychometrika* **14**, 95–116 (1949).

[3] Watts, D. J. & Strogatz, S. H. Collective dynamics of "small-world" networks. *Nature* **393**, 440–442 (1998).

[4] Walsh, T. Search in a small world. In Dean, T. (ed.) *Proceedings of the 16th International Joint Conference on Artificial Intelligence* (Morgan Kaufmann, 1999).

[5] Latora, V. & Marchiori, M. Efficient behavior of small-world networks. *Physical Review Letters* **87** (2001).

[6] Barabási, A. & Albert, R. Emergence of scaling in random networks. *Science* **286**, 509–512 (1999).
